# Social support ecosystems for adolescent physical activity: a mixed-methods evaluation framework and empirical evidence from China

**DOI:** 10.3389/fpubh.2026.1808568

**Published:** 2026-07-15

**Authors:** Heng Luo, Zelong Chen

**Affiliations:** 1 School of Physical Education, Xiangnan University, Chenzhou, China; 2 Xiangsihu College of Guangxi Minzu University, Guangxi University for Nationalities, Nanning, China

**Keywords:** adolescents, evaluation system, health of adolescents, physical health promotion, social support

## Abstract

**Purpose:**

This study aimed to establish an evaluation system for social support in the field of adolescent physical health promotion and conduct empirical research, so as to provide a theoretical basis for optimizing the social support environment for adolescents’ physical activity.

**Methods:**

This study employed the Delphi method and the Analytic Hierarchy Process (AHP) to establish the evaluation index system. Subsequently, an empirical application was conducted from March to July 2025, involving 940 participants across six regions in Hunan Province. The Fuzzy Comprehensive Evaluation (FCE) method was utilized to analyze the data.

**Results:**

The social support evaluation system for adolescent physical health promotion consists of 4 primary indicators (government support, school support, family support, and community support), 19 secondary indicators, and 54 tertiary indicators. Among the primary indicator weights, government support had the largest weight (0.3764), followed by school support (0.2885), family support (0.2442), and community support (0.0909). For social support of adolescent physical health promotion in Hunan Province, government support scored the highest (87.56), reaching a “good” level; school support (80.82) and family support (75.40) were also rated as “good”; and community support had the lowest score (60.35).

**Conclusion:**

This study underscores the significant role of social support in adolescent physical health promotion. Relatively stable social support for adolescent physical health promotion mainly includes four dimensions: government, school, family, and community. Overall, the level of social support for adolescent physical health promotion in Hunan Province is relatively positive, but structural imbalances and other shortcomings still exist. Future efforts should focus on four key stakeholders, government responsibility: bridging the gap between policy intent and implementation, school implementation: overcoming academic pressure and resource constraints, family participation: reshaping parental perceptions and health literacy, and community extension: revitalizing grassroots support systems, so as to promote the comprehensive physical and mental development of adolescents.

## 1 Introduction

Adolescent physical activity is a cornerstone for building a leading sports nation and a “Healthy China,” playing a pivotal role in national development and rejuvenation. Since the 18th National Congress, the Chinese central leadership has prioritized adolescent sports, implementing a series of policies, regulations, and action plans to encourage active participation. The Outline of the 14th Five-Year Plan for National Economic and Social Development and Vision 2035 of the People’s Republic of China explicitly mandates the in-depth implementation of adolescent physical activity promotion plans and the Healthy China initiative. Critical health issues, such as obesity, persist despite policy interventions. Currently, the compliance rate for physical activity among Chinese adolescents is merely 8.2%, with average daily moderate-to-vigorous physical activity (MVPA) amounting to less than 30 min (1). Projections indicate that the prevalence of overweight and obesity among Chinese children and adolescents could rise to 28% by 2030, affecting approximately 49.5 million individuals. Furthermore, 14.8% of the adolescent population in China faces varying degrees of depression risk, with an overall detection rate of mental health issues at 18.9%, reflecting an upward trend and an onset at younger ages (2). Data from a global survey of physical activity levels among adolescents aged 13–15 years across 105 countries indicate that the overall physical activity status of adolescents worldwide is unsatisfactory, with 80.3% failing to meet the daily recommendation of 60 min of moderate-to-vigorous physical activity (3). Scholars including Wohlgemuth (4), Behjat (5), Hamar (6), and Djordjevic-Nikic (7) have separately investigated sports participation among adolescents in Brazil, Iran, Hungary, and Yugoslavia using questionnaire-based methods. Their findings consistently reveal common challenges, including insufficient sports participation and low physical activity levels among adolescents in these nations.

Social support is widely recognized as a critical determinant of health promotion and a key variable influencing health behaviors and health outcomes (8). Scholars such as Caplan and Thoits (9, 10) adopted a bidirectional interactive perspective to dissect the multifaceted functions of social support. They posited that social support not only furnishes individuals with necessary assistance to meet practical needs but also fosters self-awareness and personal development. In the domain of adolescent physical health promotion, social support can be defined as the process by which adolescents obtain material or tangible help from the external environment, thereby enhancing physical and mental health as well as social adaptability through physical activity (11). Extensive research has explored the link between social support and sports participation, with a general consensus that social support serves as one of the important predictors of sports participation and is significantly positively correlated with it. Abigai (12), from a cross-cultural standpoint, classified social support into formal and informal types: formal support originates from institutions such as governments and social organizations, whereas informal support derives from close relational networks including families and friends. Kurc (13) documented that sufficient social support and active investment in sports resources by schools and communities can markedly stimulate adolescents’ participation enthusiasm and effectively elevate their overall activity levels. Bunke (14) further noted that social support from sports clubs is the most influential, followed by support from recreational activities, campus environments, and family backgrounds, highlighting the hierarchical effects of social support from different sources on adolescent sports participation.

Karen (15) conducted a study among 30,501 adolescents aged 6–17 years and found that parental emotional support is significantly associated with children’s physical and social activities, although this effect is relatively weaker among children with autism spectrum disorder. Consistently, King (16) confirmed that social support significantly shapes adolescents’ perceptions and engagement in physical activity. Adolescents receiving higher levels of support from parents and peers exhibit more frequent physical exercise days, further validating the positive role of social support.

Social ecological theory emphasizes that the focus of health promotion should not be confined to individual health behaviors, but extended to dynamic interactions between individuals, between individuals and groups, and between individuals and the broader social environment, advocating integrated strategies of behavior change and environmental optimization (17). Stokols’ social ecological model has transformed the paradigm of exploring adolescent physical activity promotion from a single biological perspective, highlighting the impact of the social-ecological environment on individual behavior change. It is generally acknowledged in academic circles that, in adolescent physical health promotion, governments, schools, families, communities, social organizations and institutions, sports associations and groups can all provide support for adolescents’ sports participation. Nevertheless, adolescents lack independent living capacity, financial independence, autonomous judgment and awareness of power expression, and have relatively limited contact with professional social groups, enterprises or organizations. Consequently, they tend to seek assistance and support from closely related persons and institutions in their immediate surroundings during physical exercise. Meanwhile, personal support is characterized by considerable instability and weak resource provision. Accordingly, based on Bronfenbrenner’s social ecological systems theory, this study subdivides social support into four dimensions: governmental support, school support, community support, and family support.

Despite existing research findings, the social support environment for adolescent physical health promotion in China still presents obvious shortcomings (18). Severe deficits exist in the provision of family and community support (19), accompanied by practical dilemmas such as weakened policy effectiveness and imbalanced supply and demand of sports resources (20). Moreover, the absence of a scientific evaluation system and mechanism for social support in adolescent physical health promotion makes it difficult to effectively guarantee and implement relevant support measures and their intensity, leaving the efficacy of social support far from maximized. Most academic research on social support evaluation adopts the Perceived Social Support Scale (PSSS) and Social Support Rating Scale (SSRS), or slightly adapted versions thereof. However, social support measurement should be tailored to specific groups and contextual scenarios. For adolescent physical health promotion, social support mainly derives from government, schools, families and communities, yet current adapted scales fail to cover these key providers, rendering comprehensive assessment unfeasible.

In view of the above, this study conducts a systematic investigation into social support for adolescent physical health promotion by drawing on social support theory, systems theory, social ecological theory and other relevant theories. It aims to construct an evaluation index system of social support for adolescent physical health promotion and carry out empirical research, so as to provide a scientific assessment tool for social support in this field and propose targeted optimization strategies. By gradually improving the social support framework for adolescent physical health promotion across multiple dimensions, this study will further advance the theoretical and practical development of social support for adolescent physical health promotion.

## 2 Methodology

### 2.1 Participants and data collection

This study selected six representative regions in Hunan Province—Changsha, Xiangtan, Zhuzhou, Hengyang, Huaihua, and Xiangxi Autonomous Prefecture—as the survey areas. A stratified random cluster sampling method was employed to distribute questionnaires to four distinct groups: (1) officials and staff from sports bureaus, education bureaus, and sub-district offices; (2) school administrators and physical education (PE) teachers in primary and secondary schools; (3) community leaders (secretaries, directors) and sports officers; and (4) parents of adolescents.

Data were collected through both paper-based questionnaires and the online platform Wenjuanxing. A total of 940 questionnaires were retrieved. After excluding invalid responses, 887 valid questionnaires remained, comprising 62 for government support, 221 for school support, 192 for community support, and 412 for parental support. All participants provided written informed consent, and the study protocol was approved by the Ethics Committee of Hunan University of Technology.

### 2.2 Questionnaire survey

The indicators within the constructed evaluation index system for social support in adolescent physical health promotion were categorized and operationalized into corresponding questionnaire items. Four specific questionnaires were designed targeting the four stakeholders (government, school, community, and family). The draft questionnaires were submitted to an expert panel for review; 95% of the experts rated the content validity as high. The reliability of the questionnaires was assessed using the test–retest method. Two surveys administered 14 days apart demonstrated a high degree of correlation (r = 0.90, *p* < 0.05). To ensure the reliability and validity of the four sets of questionnaire data, this study separately examined the reliability and validity of the governmental support questionnaire, school support questionnaire, community support questionnaire, and family support questionnaire. The reliability test results showed that the Cronbach’s *α* coefficients of the governmental support, school support, community support, and family support questionnaires were 0.842, 0.887, 0.861, and 0.806, respectively, all above 0.800, indicating that all four questionnaires possessed satisfactory internal consistency and high reliability. The validity test results revealed that the KMO values of the four questionnaires were 0.781, 0.872, 0.836, and 0.724, respectively, all greater than 0.700, suggesting that the sample data were suitable for factor analysis. The Bartlett’s test of sphericity yielded a significance level of 0.000 for all four questionnaires, which was less than 0.05, demonstrating significant correlations among the questionnaire indicators and good structural validity. Further analysis indicated that the factor loadings of all items in each questionnaire exceeded 0.500, meaning that each item could effectively reflect the content of its corresponding dimension.

### 2.3 Delphi method

A panel of 13 experts was selected from three domains: sociology of sport, adolescent physical health promotion, and youth sports management (specific details are provided in the Table 1). The evaluation index system was progressively constructed and refined through 3 rounds of expert consultation. In this study, 13 experts were selected from the fields of sports sociology, adolescent physical health promotion, and adolescent sports management practice to participate in three rounds of Delphi consultation. The first round was mainly used to screen and revise the preliminary indicators. The second round aimed to evaluate the importance of indicators and examine the convergence of expert opinions. The third round was conducted to construct the judgment matrix for the analytic hierarchy process (AHP) and determine indicator weights. This study employed the Delphi method and analytic hierarchy process (AHP) to construct the social support evaluation indicator system for adolescent physical health promotion. The specific procedures were as follows: preliminary evaluation indicators were established through qualitative analysis; the indicator system was revised in the first round of expert consultation; statistical tests were performed on the indicator system in the second round of expert consultation; indicator weights were determined using the analytic hierarchy process in the third round of expert consultation; and finally, the social support evaluation indicator system for adolescent physical health promotion was formally established.

**Table 1:** Summary of expert basic information (*N* = 13).

No.	Name	Affiliation	Title/Position
1	YZH	East China Normal University	Professor
2	WSQ	Hunan University of Technology	Professor
3	GDH	Hunan Normal University	Professor
4	HCW	Hunan University of Technology	Professor
5	LMY	Wuhan Sports University	Professor
6	ZLP	Hunan University of Technology	Professor
7	YCD	Nanjing Sport Institute	Professor
8	JYQ	Hunan Normal University	Professor
9	ZTC	Jishou University	Professor
10	LYY	Hunan University of Technology	Professor
11	HWB	Zhuzhou Academy of Educational Sciences	PE Researcher
12	ZYL	Zhuzhou Tianyuan District Academy of Educational Sciences	Senior Teacher
13	ZZY	Changsha Yali Experimental Middle School	Head of PE Group

### 2.4 Statistical analysis

Data analyses were performed using SPSS 25.0 software. To establish the evaluation index system, key statistical indicators—including the coefficient of variation (CV), Kendall’s coefficient of concordance (W), and asymptotic significance—were calculated to screen the indicators. Subsequently, the importance of each factor was determined through expert consultation. The Analytic Hierarchy Process (AHP) was employed to construct pairwise comparison matrices, and the weights of the evaluation indicators were derived by calculating the eigenvectors. Based on these weights, the Fuzzy Comprehensive Evaluation (FCE) method was applied to analyze the empirical data. This process involved three distinct steps: defining the factor set, establishing the fuzzy rating set, and synthesizing the evaluation results. SPSS 25.0 was employed to calculate the mean value, coefficient of variation, Cronbach’s *α* coefficient, Kendall’s W coefficient of concordance, and significance level for indicators at all levels, so as to test the reliability of the index system and the degree of expert opinion coordination. On this basis, the analytic hierarchy process (AHP) was used to construct pairwise comparison judgment matrices for indicators at the same level, and the weight vector, maximum eigenvalue λmax, consistency index (CI), and consistency ratio (CR) were computed. A judgment matrix was deemed to possess satisfactory consistency when CR < 0.10. Furthermore, the fuzzy comprehensive evaluation method was applied to assess the level of social support for adolescent physical health promotion, following the procedures of defining the factor domain, establishing the rating grade domain, constructing the fuzzy relation matrix, determining the weight vector, synthesizing the comprehensive evaluation vector, and calculating the comprehensive score.

### 2.5 Construction and determination of the evaluation index system

#### 2.5.1 Construction of the preliminary evaluation index system

This study adopted a qualitative research design. Participants were selected via purposive sampling from Changsha and Zhuzhou in Hunan Province to conduct in-depth interviews. The sample consisted of 8 adolescents, 12 parents, 6 physical education (PE) teachers, 4 school administrators, 8 government officials (from education and sports sectors), and 12 community staff members. The interviews generated approximately 186,000 words of raw textual data. NVivo 14 software was utilized for data cleaning, screening, and three-level coding. This process yielded a preliminary evaluation index system consisting of 4 primary indicators, 19 sary indicators, and 55 tertiary indicators.

#### 2.5.2 Determination of the final evaluation index system

*Round 1 expert consultation*: Based on the preliminary index system, the first round of expert consultation was conducted using a questionnaire. For each indicator, experts selected one of three options: “Retain,” “Modify,” or “Delete.” An open-ended section was included at the end of the questionnaire for experts to suggest additions or removals. After sorting and analyzing the returned questionnaires, it can be concluded that the entry rate of the four first-level indicators reached 100%. Experts fully affirmed the evaluation of social support for adolescent physical health promotion from the four dimensions of government, school, family, and community; therefore, no revision was required for these indicators. Experts put forward the following opinions on the second-level indicators:

“Public sports venue service facilities” was revised to “youth sports venues and facilities”; “Adolescent sports events” was revised to “adolescent sports event system”; “Adolescent sports funding” was revised to “adolescent sports funding support”; “Adolescent sports policy” was revised to “adolescent sports policy support.” The indicator “Dissemination of sports and health knowledge” was not on the same dimension as other secondary indicators, and experts suggested revising it to “Adolescent sports activities” or other appropriate alternatives. “Linkage with families and communities” was revised to “Sports linkage with families and communities.”

In response to the above expert opinions, adjustments to the secondary indicators were made as follows:

“Sports and health knowledge dissemination” was deleted. “Public sports venue service facilities” was revised to “adolescent sports venues and facilities,” and “community sports venues and facilities” was added. “Adolescent sports events” was revised to “adolescent sports event system.” “Adolescent sports funding” was revised to “adolescent sports funding support.” “Adolescent sports policy” was revised to “adolescent sports policy support.”

Experts put forward the following opinions on the formulation of tertiary indicators:

Experts raised the following comments on tertiary indicators: The item “availability of venues and facilities” is similar to the previous indicator; it is suggested to revise it to “advancement.” Regarding “completeness of adolescent sports event structure”: the sports event structure is fixed and not subject to artificial alteration; it is suggested to replace it with “number of events held.” The expression “comprehensiveness of adolescent sports funding” is not commonly used; it is suggested to revise it to “diversification of funding channels.” The expression “stability of policy content” is inaccurate. It is suggested to revise “systematization degree of sports competitions” to “diversification degree of sports competition implementation.” It is suggested to revise “completeness of home sports assignments” to “completion status of home sports assignments.” It is suggested to revise “comprehensiveness of sports funding budget” to “rationality of sports funding.” The item “stability of teachers’ sports activities” is highly homogeneous with the next indicator; it is suggested to revise it to “strength of campus sports culture.” Some tertiary indicators under certain secondary indicators are not comprehensive and detailed enough. For instance, under the secondary indicator “family sports consumption,” the item “satisfaction degree of family sports equipment with sports needs” should include family consumption investment in sports events and activities. The expressions of several indicators are considered inaccurate and require revision, including “effectiveness of institutional implementation,” “stability of teachers’ sports activities,” “stability of teachers’ encouragement and accompaniment in physical exercise,” “equality of relationships among school, family and community,” and “satisfaction degree of family sports equipment with sports needs.”

In response to the above expert opinions:

The tertiary indicators were adjusted as follows: “Availability of venues and facilities” was revised to “Diversity of venues and facilities”; “Completeness of adolescent sports event structure” was revised to “Number of adolescent sports events held”; “Comprehensiveness of adolescent sports funding” was revised to “Diversified channels of adolescent sports funding”; “Stability of adolescent sports policy content” was revised to “Pragmatism of adolescent sports policy content”; “Systematization degree of sports competitions” was revised to “Diversification degree of sports competition implementation”; “Completeness of home sports assignments” was revised to “Completion status of home sports assignments”; “Comprehensiveness of the funding budget” was revised to “Rationality of the funding budget”; “Effectiveness of funding utilization” was revised to “Utilization status of sports funding”; “Effectiveness of institutional implementation” was revised to “Implementation status of the institution”; “Stability of teachers’ sports activities” was revised to “Construction status of campus sports culture”; “Stability of teachers’ encouragement and accompaniment in physical exercise” was revised to “Status of teachers’ encouragement and accompaniment in physical exercise”; “Stability of peers’ encouragement and accompaniment in physical exercise” was revised to “Status of peers’ encouragement and accompaniment in physical exercise”; “Equality of relationships among school, family and community” was revised to “Establishment status of an adolescent sports network integrating school, family and community”; “Stability of community sports guidance” was revised to “Status of community sports guidance”; “Stability of community sports activities” was revised to “Implementation status of community sports activities”; “Configuration status of community sports venues and facilities” and “Spatial distribution status of community sports venues and facilities” were added; “Satisfaction degree of family sports equipment with sports needs” was revised to “Family consumption investment in sports events and activities”; “Stability of family sports activities” was revised to “Implementation status of family sports activities.”

*Round 2 expert consultation*: The revised secondary and tertiary indicators were compiled into a 5-point Likert scale (ranging from 5 = “Very Important” to 1 = “Very Unimportant”). Experts were also provided with explanatory notes regarding the modifications. The results indicated that the mean scores for all secondary and tertiary indicators exceeded 4.0 (with a minimum threshold of 3.5), and the coefficients of variation (CV) were all below 0.25, meeting the retention criteria. Therefore, the final evaluation index system of social support for adolescent physical health promotion was formulated, which is composed of 4 first-level indicators, 19 second-level indicators and 54 third-level indicators.

The expert consultation results showed that the Cronbach’s *α* coefficients of the first-level, second-level, and third-level indicators were 0.924, 0.815, and 0.989, respectively, all above the acceptable level; the Kendall’s W coefficients of concordance were 0.325, 0.407, and 0.227, respectively, and all significance levels met statistical requirements, indicating good consistency among expert opinions (Table 2).

**Table 2:** Results of reliability and coordination tests for Delphi expert consultation.

Level	Cronbach’s α	Kendall’s W	χ^2^	df	P
First-level Indicators	0.924	0.325	12.667	3	0.005
Second-level Indicators	0.815	0.407	95.235	18	0.000
Third-level Indicators	0.989	0.227	156.630	53	0.000

*The third round of expert consultation*: Experts conducted pairwise comparisons of indicators at the same level using the Saaty 1–9 scale method to construct judgment matrices, and calculated the maximum eigenvalue, weight vector, consistency index (CI), and consistency ratio (CR). The results demonstrated that the consistency ratios of all judgment matrices at each level were less than 0.10, indicating satisfactory consistency of the judgment matrices. It should be noted that the weights presented in this paper are the comprehensive weights synthesized from expert group judgments via the analytic hierarchy process (AHP). Therefore, the judgment matrices shown in the main text are mainly used to illustrate the weight formation process and the results of the consistency test.

*Judgment matrix and consistency test*: Given that different indicators exert varying degrees of influence on the level of social support for adolescent physical health promotion, referring to the research framework of AHP-fuzzy comprehensive evaluation, this paper adopts the 1–9 scale method to conduct pairwise comparison of indicators at the same level, constructs judgment matrices, and calculates the weight vector, maximum eigenvalue λmax, consistency index CI, and consistency ratio CR. A judgment matrix is considered to have satisfactory consistency when CR < 0.10. For the convenience of presentation, the four primary indicators in this paper are denoted as B1 (government support), B2 (school support), B3 (community support), and B4 (family support).

Calculated from Table 3, λmax = 4.0458, CI = 0.0153, RI = 0.900, CR = 0.0170 < 0.10, indicating that the judgment matrix of first-level indicators passes the consistency test. Combined with the results of expert consultation and weight calculation, the weights of the first-level indicators are 0.3764 for government support, 0.2885 for school support, 0.0909 for community support, and 0.2442 for family support, respectively.

**Table 3:** Judgment matrix of first-level indicators B1–B4.

First-level Indicators	B1	B2	B3	B4
B1	1	1	4	2
B2	1	1	3	1
B3	1/4	1/3	1	1/3
B4	1/2	1	3	1

Calculated from Table 4, λmax = 4.0104, CI = 0.0035, CR = 0.0038 < 0.10, which indicates that the judgment matrix of secondary indicators under government support passes the consistency test. The weights of the secondary indicators under government support are as follows: C11 Adolescent sports venues and facilities 0.1409, C12 Adolescent sports event system 0.1409, C13 Adolescent sports funding support 0.2628, and C14 Adolescent sports policy support 0.4554.

**Table 4:** Judgment matrix of secondary indicators under government support.

Government Support	C11	C12	C13	C14
C11	1	1	1/2	1/3
C12	1	1	1/2	1/3
C13	2	2	1	1/2
C14	3	3	2	1

Calculated from Table 5, indicating that the judgment matrix of secondary indicators under school support passes the consistency test. The weights of the secondary indicators under school support are as follows: C21 School sports venues and equipment 0.1831, C22 School sports activities 0.1024, C23 School physical education teachers 0.1831, C24 School sports funds 0.1024, C25 School sports rules and regulations 0.0916, C26 School sports atmosphere 0.1542, C27 Sports linkage with family and community 0.1831.

**Table 5:** Judgment matrix of secondary indicators under school support.

School support	C21	C22	C23	C24	C25	C26	C27
C21	1	2	1	2	2	1	1
C22	1/2	1	1/2	1	1	1	1/2
C23	1	2	1	2	2	1	1
C24	1/2	1	1/2	1	1	1	1/2
C25	1/2	1	1/2	1	1	1/2	1/2
C26	1	1	1	1	2	1	1
C27	1	2	1	2	2	1	1

Calculated from Table 6, λmax = 5.0278, CI = 0.0070, CR = 0.0062 < 0.10, which indicates that the judgment matrix of secondary indicators under community support passes the consistency test. The weights of the secondary indicators under community support are as follows: C31 Community sports guidance 0.0824, C32 Community sports organization 0.0866, C33 Community sports activities 0.1507, C34 Community sports funds 0.2537, C35 Community sports venues and facilities 0.4266.

**Table 6:** Judgment matrix of secondary indicators under community support.

Community support	C31	C32	C33	C34	C35
C31	1	1	1/2	1/3	1/5
C32	1	1	1/2	1/3	1/4
C33	2	2	1	1/2	1/3
C34	3	3	2	1	1/2
C35	5	4	3	2	1

Calculated from Table 7, λmax = 3.0536, CI = 0.0268, CR = 0.0462 < 0.10, indicating that the judgment matrix of secondary indicators under family support passes the consistency test. The weights of the secondary indicators under family support are as follows: C41 Family sports atmosphere 0.5278, C42 Family sports consumption 0.1396, C43 Family sports activities 0.3325.

**Table 7:** Judgment matrix of secondary indicators under family support.

Family support	C41	C42	C43
C41	1	3	2
C42	1/3	1	1/3
C43	1/2	3	1

The CR values of judgment matrices at all levels are less than 0.10, which demonstrates satisfactory consistency of expert judgments, and the obtained weights are applicable to the subsequent fuzzy comprehensive evaluation. The weights of tertiary indicators are calculated by the same method and summarized in the indicator weight table. After acquiring the local weights of indicators at each level, the comprehensive weights of tertiary indicators relative to the target layer are further calculated. The calculation formula of comprehensive weight is as follows: ${\mathrm{CW}}_{\mathrm{ijk}}=W_{i}\times W_{\mathrm{ij}}\times W_{\mathrm{ijk}.}$

Where ${\mathrm{CW}}_{\mathrm{ijk}}$ represents the comprehensive weight of the k-th tertiary indicator under the j-th secondary indicator of the i-th primary indicator relative to the target layer, $W_{i}$ represents the weight of the primary indicator, $W_{\mathrm{ij}}$ represents the local weight of the secondary indicator, and $W_{\mathrm{ijk}}$ represents the local weight of the tertiary indicator (Table 8).

**Table 8:** Weights of the evaluation index system for social support in adolescent physical health promotion.

Primary indicators (Weight)	Secondary indicators (Weight)	Tertiary indicators (Weight)	Global weight	Overall rank
Government support (0.3764)	Youth sports venues & facilities (0.1409)	Standard compliance of venues & facilities (0.3165)	0.017274	24
		Diversity of venues & facilities (0.2443)	0.013333	30
		Accessibility of venues & facilities (0.4392)	0.023971	13
	Youth sports event system (0.1409)	Stability of youth sports event organization (0.3969)	0.020586	18
		Frequency of youth sports events (0.2699)	0.013999	28
		Coverage of youth sports events (0.3333)	0.017288	23
	Youth sports funding support (0.2628)	Proportion of youth sports funding in total budget (0.3163)	0.032681	8
		Diversity of funding channels (0.1850)	0.019115	19
		Efficiency of youth sports fund usage (0.4987)	0.051527	2
	Youth sports policy support (0.4554)	Comprehensiveness of policy plans (0.1138)	0.018963	21
		Pragmatism of policy content (0.2534)	0.042225	6
		Effectiveness of policy implementation (0.6328)	0.105445	1
School support (0.2885)	School sports venues & equipment (0.1831)	Standard compliance of school venues & equipment (0.3333)	0.017664	22
		Matching of venues & equipment (0.6667)	0.035333	7
	School sports activities (0.1024)	Standard compliance of PE curriculum setting (0.2066)	0.007152	39
		Teaching quality of PE curriculum (0.1843)	0.006380	41
		Intensity of “Grand Recess” activities (0.1434)	0.004965	44
		Stability of extracurricular training (0.0888)	0.003074	50
		Diversity of sports competitions (0.1324)	0.004584	46
		Richness of sports club activities (0.1997)	0.006914	40
		Completion of family sports homework (0.0448)	0.001551	54
	School sports faculty (0.1831)	Compliance of PE student-teacher ratio (0.3750)	0.019095	20
		Professional level of PE faculty (0.6250)	0.031825	10
	School sports funding (0.1024)	Rationality of sports budget (0.3333)	0.010346	33
		Usage of sports funding (0.6667)	0.020696	17
	School sports regulations (0.0916)	Comprehensiveness of regulations (0.1667)	0.004420	47
		Implementation of regulations (0.8333)	0.022093	14
	School sports atmosphere (0.1542)	Importance attached by school leaders & teachers (0.3080)	0.012742	31
		Construction of campus sports culture (0.2097)	0.008675	36
		Teacher encouragement & accompaniment (0.2798)	0.011576	32
		Peer encouragement & accompaniment (0.2025)	0.008378	38
	Linkage with family & community (0.1831)	Clarity of relationships (School-Family-Community) (0.2640)	0.013466	29
		Establishment of network (School-Family-Community) (0.1977)	0.010084	34
		Synergy between School, Family & Community (0.5384)	0.027462	12
Community support (0.0909)	Community sports guidance (0.0824)	Professionalism of community instructors (0.2655)	0.001904	52
		Scientific nature of guidance methods (0.2352)	0.001687	53
		Ratio of community instructors (0.4993)	0.003581	49
	Community sports organization (0.0866)	Completeness of community sports organization structure (0.6923)	0.006192	42
		Vitality of community sports organizations (0.3077)	0.002752	51
	Community sports activities (0.1507)	Implementation of community sports activities (0.3866)	0.005700	43
		Standardization of community sports activities (0.2809)	0.004142	48
		Popularity of community sports activities (0.3325)	0.004902	45
	Community sports funding (0.2537)	Per capita sports funding investment (0.3750)	0.008495	37
		Proportion of sports funding in total budget (0.6250)	0.014158	27
	Community sports venues & facilities (0.4266)	Configuration of community venues & facilities (0.4286)	0.016024	26
		Spatial distribution of community venues & facilities (0.5714)	0.021363	15
Family support (0.2442)	Family sports atmosphere (0.5278)	Stability of parents’ participation in sports (0.0712)	0.009111	35
		Importance attached by parents to sports (0.2412)	0.030864	11
		Stability of parental guidance (0.3501)	0.044799	4
		Stability of parental encouragement & accompaniment (0.3376)	0.043200	5
	Family sports consumption (0.1396)	Family investment in sports events & activities (0.5625)	0.020728	16
		Stability of family sports consumption (0.4375)	0.016122	25
	Family sports activities (0.3325)	Implementation of family sports activities (0.4091)	0.032478	9
		Intensity of family sports activities (0.5909)	0.046911	3

## 3 Results

### 3.1 Determination of evaluation grades

Based on the questionnaire design and incorporating expert recommendations, this study adopted a 5-point Likert scale. The evaluation set was operationalized into five response options, denoted as V = {V1, V2, V3, V4, V5}. These correspond to the linguistic variables {Very Important, Important, Average, Less Important, Not Important}, which were assigned the values {5, 4, 3, 2, 1}, respectively. For quantitative analysis, the score vector was assigned as V = [100, 80, 60, 40, 20] (Table 9).

**Table 9:** Overall evaluation criteria for social support in adolescent physical health promotion.

Evaluation grade	V1 (Very Good)	V2 (Good)	V3 (Average)	V4 (Poor)	V5 (Very Poor)
Score range	100–90	89–75	74–55	54–40	< 39

### 3.2 Data collection

The survey was conducted in six representative regions of Hunan Province: Changsha, Xiangtan, Zhuzhou, Hengyang, Huaihua, and Xiangxi Autonomous Prefecture. Questionnaires were distributed to four specific groups of stakeholders within these regions: (1) Government officials and staff from sports bureaus, education bureaus, and sub-district offices; (2) School administrators and physical education (PE) teachers in primary and secondary schools; (3) Community leaders (secretaries, directors) and sports officers; (4) Parents of adolescents.

A total of 940 questionnaires were retrieved through both paper-based forms and the online platform Wenjuanxing. To ensure data quality, invalid questionnaires were excluded based on the following criteria: (1) respondents who self-reported being “very unfamiliar” or “not very familiar” with school sports work and student physical health; (2) response times that were excessively short (indicating a lack of serious engagement); and (3) responses with identical scores for all items (straight-lining).

After data cleaning, 887 valid questionnaires were obtained, yielding an effective response rate. The validity breakdown is as follows: 62 government support questionnaires, 221 school support questionnaires, 192 community support questionnaires, and 412 parental support questionnaires (Table 10).

**Table 10:** Distribution of sample regions (*n* = 887).

Region	Government	School	Family	Community	Total
Changsha	11	42	89	36	178
Zhuzhou	14	53	105	43	215
Xiangtan	10	31	56	29	126
Hengyang	10	35	60	33	138
Huaihua	9	28	54	26	117
Xiangxi	8	32	48	25	113

### 3.3 Data processing

Given the specificity and complexity of social support for adolescent physical health promotion, along with the comparative advantages of various comprehensive evaluation methods, this study employed the Fuzzy Comprehensive Evaluation (FCE) method. Following the determination of indicator weights via AHP, the FCE procedure was conducted through the following steps:

(1) Determination of the factor set (universe of discourse)

Let $U=\{u_{1},u_{2,}\ldots ,u_{m}\}$ denote the set of all indicators that characterize the evaluation object.

(2) Determination of the evaluation grade set

Let $V=\{v_{1},v_{2,}\ldots ,v_{m}\}$ represent the set of evaluation grades (comments) characterizing the indicators. While this parameter can be adjusted based on the specific index system, it is typically divided into 3 to 5 levels. In this study, the evaluation grades are divided into 5 levels, with each level corresponding to a specific fuzzy subset.

(3) Establishment of the Fuzzy Relation Matrix (R)

The fuzzy relation matrix $R$, constructed based on the evaluation indicators and the evaluation object, is expressed as:


$$R={\left(r_{\mathrm{ij}}\right)}_{m\times n}=[\begin{array}{cccc} r_{11} & r_{12} & \cdots & r_{1n}\\ r_{21} & r_{22} & r_{23} & r_{2n}\\ \vdots & \vdots & \ddots & \vdots \\ r_{m1} & r_{m2} & \cdots & r_{\mathrm{mn}} \end{array}]$$

(4) Determination of the Weight Vector Let the weight vector for the system be $A=\{a_{1},a_{2},\ldots a_{n}\}$ . In this set, $a_{i}$ represents the membership degree of the $u_{i}$ indicator to the evaluation target system. In the process of determining the weight vector, the weights derived from AHP are applied to the fuzzy matrix to calculate the weight levels of each indicator. Subsequently, the weight coefficients are calculated and normalized. The calculation method is as follows:

$$\sum_{i=1}a_{i}=1,a_{i}\geq 0,i=1,2,\ldots ,n$$

(5) Synthesis of the Fuzzy comprehensive evaluation result vector the evaluation result vector B is obtained through the matrix composition operation:

$$B=(a_{1},a_{2},\ldots a_{p})[\begin{array}{cccc} r_{11} & r_{12} & \cdots & r_{1m}\\ r_{21} & r_{22} & r_{23} & r_{2m}\\ \vdots & \vdots & \ddots & \vdots \\ r_{p1} & r_{p2} & \cdots & r_{\mathrm{pn}} \end{array}]=(b_{1},b_{2},\ldots ,b_{m})$$

(6) Analysis of Fuzzy comprehensive evaluation results in practical applications, the Maximum Membership Principle is often used to interpret the calculation results of fuzzy comprehensive evaluation. However, given that the data collection in model construction relies on subjective evaluation, solely relying on the maximum membership might lead to unreasonable conclusions. To address this, the Weighted Average Method was selected to perform a quantitative analysis of the final evaluation results, ensuring a more robust interpretation of the membership grades.

Through the analysis and processing of the valid questionnaires, the following results were obtained:


$$S=(a_{1},a_{2},\ldots a_{p})[\begin{array}{cccc} b_{11} & b_{12} & \cdots & b_{1m}\\ b_{21} & b_{22} & b_{23} & b_{2m}\\ \vdots & \vdots & \ddots & \vdots \\ b_{p1} & b_{p2} & \cdots & b_{\mathrm{pn}} \end{array}]=(s_{1},s_{2},\ldots ,s_{m})$$

Based on the analysis of the valid questionnaires, the evaluation results were derived.

Evaluation vector for government support:


$$\begin{array}{l} B_{1}=(0.145,0.1378,0.2745,0.4427)\\ \left[\begin{array}{rrrrr} 0.557415 & 0.353691936 & 0.065681 & 0.016129 & 0.007084\\ 0.562524 & 0.3559 & 0.061192 & 0.020484 & 0\\ 0.54 & 0.30476129 & 0.101834 & 0.045361 & 0.008044\\ 0.547774 & 0.324219355 & 0.089213 & 0.0245 & 0.014294 \end{array}\right]\\ =(0.549071,0.327517233,0.085404,0.028459,0.009563) \end{array}$$

Evaluation vector for school support:


$$\begin{array}{l} B_{2}=(0.1837,0.12,0.1765,0.1076,0.0919,0.1434,0.1768)\\ \left[\begin{array}{rrrrr} 0.351433 & 0.333333937 & 0.289594 & 0.024131 & 0.001508\\ 0.356097 & 0.335228054 & 0.286533 & 0.020404 & 0.001738\\ 0.378394 & 0.330316742 & 0.286765 & 0.004525 & 0\\ 0.363499 & 0.337858371 & 0.265459 & 0.033184 & 0\\ 0.361991 & 0.343137104 & 0.287331 & 0.007541 & 0\\ 0.36522 & 0.329400452 & 0.297501 & 0.006612 & 0.001266\\ 0.358396 & 0.326671946 & 0.305634 & 0.009398 & 0 \end{array}\right]\\ =(0.362193,0.332641201,0.289863,0.014553,0.000667) \end{array}$$

Evaluation vector for community support:


$$\begin{array}{l} B_{3}=(0.0789,0.0984,0.1622,0.2492,0.4113)\\ \left[\begin{array}{rrrrr} 0.035879 & 0.2830323 & 0.377263 & 0.284038 & 0.019788\\ 0.064904 & 0.2832536 & 0.377804 & 0.232773 & 0.041266\\ 0.041799 & 0.2845661 & 0.373617 & 0.247529 & 0.052489\\ 0.044922 & 0.2714844 & 0.371745 & 0.288411 & 0.023437\\ 0.046129 & 0.2574417 & 0.365327 & 0.304316 & 0.026786 \end{array}\right]\\ =(0.046165,0.2698997,0.370441,0.282502,0.030993) \end{array}$$

Evaluation vector for family support:


$$\begin{array}{l} B_{4}=(0.524,0.1509,0.3251)\\ \left[\begin{array}{rrrrr} 0.187971 & 0.4180359 & 0.365334 & 0.027588 & 0.001171\\ 0.193265 & 0.4244539 & 0.366201 & 0.035194 & 0.002124\\ 0.176743 & 0.4233231 & 0.373235 & 0.025265 & 0.001434 \end{array}\right]\\ =(0.18512,0.4207232,0.368034,0.02798,0.0014) \end{array}$$

Construction of the Fuzzy Membership matrix consequently, the fuzzy membership matrix (R) for the primary indicators is obtained as follows:


$$R=[\begin{array}{rrrrr} 0.549071 & 0.3275172 & 0.085404 & 0.028459 & 0.009563\\ 0.362193 & 0.3326412 & 0.289863 & 0.014553 & 0.000667\\ 0.046165 & 0.2698997 & 0.370441 & 0.282502 & 0.030993\\ 0.18512 & 0.4207232 & 0.368034 & 0.02798 & 0.0014 \end{array}]$$

Overall evaluation calculation the weight vector for the primary indicators, as previously determined via the Analytic Hierarchy Process (AHP), is:


W=(0.3764,0.2885,0.0909,0.2442)

By multiplying the weight vector of the primary indicators by their corresponding fuzzy membership matrix, the overall evaluation vector (B) is derived:


B=WR=(0.360565,0.346519,0.239318,0.047423,0.006951)

Final score calculation based on the target layer evaluation vector (B) and the grade score vector (V), the final evaluation score (F) is calculated using the formula:


$$F=VB^{T}=[100\;\;80\;\;60\;\;40\;\;20][\begin{array}{r} 0.360565\\ 0.346519\\ 0.239318\\ 0.047423\\ 0.006951 \end{array}]=80.1731$$

Based on the principle of maximum membership and the previously established evaluation criteria, the overall level of social support for adolescent physical health promotion in Hunan Province is categorized as “Good” (or “Satisfactory”). The detailed evaluation results for indicators at all hierarchical levels are presented in Table 9.

## 4 Analysis and discussion

An evaluation index system serves not only to objectively measure the current status of the evaluation object but, more importantly, to diagnose deficiencies and forecast development trends. Based on the empirical data from Hunan Province, this study reveals a hierarchical structure of social support—ranked Government > School > Family > Community. This section analyzes the underlying mechanisms of these disparities and proposes evidence-based pathways for optimizing the social support system for adolescent physical health promotion (Table 11).

**Table 11:** Evaluation results for social support in adolescent physical health promotion.

Primary indicators	Score	Secondary indicators	Score	Tertiary indicators	Score
Government support	87.56	Youth sports venues & facilities	88.76	Standard compliance of venues & facilities	89.68
				Diversity of venues & facilities	90.00
				Accessibility of venues & facilities	87.42
		Youth sports event system	89.22	Stability of youth sports event organization	89.03
				Frequency of youth sports events	89.68
				Coverage of youth sports events	89.03
		Youth sports funding support	86.47	Proportion of youth sports funding in total budget	87.10
				Diversity of funding channels	90.65
				Efficiency of youth sports fund usage	84.52
		Youth sports policy support	87.33	Comprehensiveness of policy plans	90.97
				Pragmatism of policy content	88.71
				Effectiveness of policy implementation	86.13
School support	80.82	School sports venues & equipment	80.18	Standard compliance of school venues & equipment	79.46
				Matching of venues & equipment	80.54
		School sports activities	80.47	Standard compliance of PE curriculum setting	80.91
				Teaching quality of PE curriculum	80.72
				Intensity of “Grand Recess” activities	79.64
				Stability of extracurricular training	80.45
				Diversity of sports competitions	81.27
				Richness of sports club activities	79.73
				Completion of family sports homework	81.09
		School sports faculty	81.65	Compliance of PE student-teacher ratio	81.54
				Professional level of PE faculty	81.72
		School sports funding	80.63	Rationality of sports budget	81.18
				Usage of sports funding	80.36
		School sports regulations	81.19	Comprehensiveness of regulations	81.27
				Implementation of regulations	81.18
		School sports atmosphere	81.01	Importance attached by school leaders & teachers	81.63
				Construction of campus sports culture	81.45
				Teacher encouragement & accompaniment	80.09
				Peer encouragement & accompaniment	80.91
		Linkage with family & community	80.69	Clarity of relationships (School-Family-Community)	80.91
				Establishment of network (School-Family-Community)	81.00
				Synergy between School, Family & Community	80.45
Community support	60.35	Community sports guidance	60.62	Professionalism of community instructors	59.58
				Scientific nature of guidance methods	59.58
				Ratio of community instructors	61.67
		Community sports organization	61.96	Completeness of community sports organization structure	61.67
				Vitality of community sports organizations	62.60
		Community sports activities	60.31	Implementation of community sports activities	61.77
				Standardization of community sports activities	62.50
				Popularity of community sports activities	56.77
		Community sports funding	60.52	Per capita sports funding investment	58.96
				Proportion of sports funding in total budget	61.46
		Community sports venues & facilities	59.84	Configuration of community venues & facilities	58.23
				Spatial distribution of community venues & facilities	61.04
Family support	75.40	Family sports atmosphere	75.29	Stability of parents’ participation in sports	75.05
				Importance attached by parents to sports	75.29
				Stability of parental guidance	75.73
				Stability of parental encouragement & accompaniment	74.85
		Family sports consumption	76.71	Family investment in sports events & activities	76.07
				Stability of family sports consumption	77.52
		Family sports activities	74.97	Implementation of family sports activities	75.15
				Intensity of family sports activities	74.85

### 4.1 Government stewardship: bridging the gap between policy intent and execution

According to the evaluation indicator system, government support mainly operates through formulating and implementing relevant policies, providing financial and technical support for the construction and maintenance of sports venues and facilities, and promoting the organization of youth sports events. In doing so, it provides adolescents with convenient and safe places for physical activity and accessible pathways for sports participation. Empirical results demonstrate that the government support dimension achieved the highest score of 87.56, approaching an excellent level. This reflects the high priority and strong commitment of the Chinese government to the healthy development of adolescents from top-level design to grassroots implementation. Existing studies consistently highlight the central role of government support in adolescent sports participation. For instance, Yuan Shijing (2021) noted that government support represents a key external driver of physical exercise among adolescents and can exert a powerful promoting effect through authoritative policies and resource guarantees. The indicator weight of 0.4554 for policy support within the evaluation system further underscores its core function. Nevertheless, in-depth analysis of tertiary indicators reveals substantial shortcomings in policy implementation and resource allocation, presenting a practical contradiction of “sound top-level design but weak grassroots delivery.”

First, administrative fragmentation and departmental barriers restrict the effectiveness of policy implementation. Sound and well-implemented sports policies can significantly expand adolescents’ sports participation opportunities and promote their long-term physical and mental health as well as social competence development (21). Under China’s vertical administrative management system, departments including sports, education, and health care mostly operate independently with vertical management modes and lack effective horizontal coordination mechanisms. Policies cannot be implemented automatically by institutional provisions alone, and the sports sector lacks cross-departmental overall planning authority, resulting in the attenuation of policy effectiveness when transmitted to the grassroots level (22).

Secondly, there is a prominent supply gap in rigid sports infrastructure. The per capita sports venue area in China is only 2.62 square meters, less than one-sixth of that of the United States (16 square meters) and Japan (19 square meters). The structural shortage of venue resources directly reduces the accessibility and convenience of adolescents’ sports activities. Limstrand (23) pointed out that the availability of venue facilities involves the rationality of opening hours and charging standards. If the opening hours fail to match adolescents’ spare time or the use cost is too high, adolescents’ participation willingness will be greatly reduced.

Lastly, the supply of normalized sports events remains inadequate. A stable event system can significantly increase participation frequency and promote physical health and social competence (24). Widely accessible events can also alleviate participation inequality, which is particularly important for adolescents in resource-limited regions (25). However, empirical data indicate that more than 90% of students have never participated in formal sports events during basic education, and only 5% have taken part in off-campus sports competitions beyond school sports meets.

Implications for optimization:

To address these gaps, the government must shift from “issuing documents” to “ensuring operability.”

(1) *Enhance policy interoperability*: Following the example of Shanghai’s Three-Year Action Plan (2024–2026), local governments should promulgate detailed implementation schemes that complement national policies like the Youth Sports Promotion Plan (26).
(2) *Establish cross-sectoral synergy*: A Joint Conference System involving sports, education, and health departments should be institutionalized to break down administrative barriers. Digital platforms integrating “Sports-Education” and “Sports-Medicine” should be developed to provide personalized health services.
(3) *External monitoring*: An independent evaluation mechanism involving third-party academic institutions should be introduced to monitor policy implementation and ensure that “soft tasks” become “hard constraints.”

### 4.2 School implementation: overcoming academic pressure and resource constraints

According to the evaluation indicator system, school support has the second-largest weight after government support, indicating that schools remain the main arena for adolescent physical health promotion. Equipped with professional physical education teachers, venues, and equipment, schools can provide standardized sports instruction and education, and organize diverse sports activities such as sports meetings and competitions, thereby helping adolescents improve their motor skills and health status. Empirical results indicate that the school support dimension achieved a score of 80.82, rated as good, representing a relatively prominent component in the current support system for adolescent physical health promotion. As the central setting for daily physical activity among adolescents, schools play an irreplaceable role in promoting physical activity, fostering exercise habits, and enhancing health literacy. Existing research confirms that the school physical education environment and related policies constitute the most effective intervention pathway for improving overall physical activity levels in adolescents (27). A well-developed provision of school physical education services can significantly increase students’ physical activity opportunities, enhance exercise persistence, and improve health outcomes. Meanwhile, the attention, attitudes, and behavioral engagement of school leaders and physical education teachers exert direct influences on students’ willingness, enthusiasm, and skill development in sports participation (28). Positive encouragement, demonstrative leadership, and regular involvement from teachers can effectively boost students’ exercise self-confidence, skill proficiency, and intrinsic motivation (29), thereby facilitating the formation of stable sports participation behaviors.

In the evaluation indicator system, the weight of school sports management regulations (0.0916) is significantly lower than that of other indicators under school support, which to some extent reflects the phenomenon that school sports management systems are not effectively implemented. Under the examination-oriented education system, persistent academic pressure crowding out physical education has become a prominent constraint. Most school administrators prioritize academic performance and pay insufficient attention to students’ physical and mental development, leaving school physical education in a long-standing passive state of “nominal emphasis, weakened implementation, and subordination to academic courses during critical periods” (30). This situation severely restricts the quality of physical education curricula and the regular implementation of extracurricular physical activities.

Implications for optimization:

(1) *Enforce the “Health First” philosophy*: The performance evaluation of school principals should explicitly include student physical health metrics to realign administrative incentives. The establishment of a supervision group to inspect the implementation of physical education courses has significantly improved the compliance rate of rules and regulations and elevated the overall teaching quality.
(2) *Curriculum reform*: Schools must transition from a “safe” but low-intensity PE model to a comprehensive “Learn, Practice, Compete, Evaluate” model. The goal is to ensure every student masters specific sports skills (31).
(3) *Home-school-community linkage*: Schools should act as the hub of a “sports life community,” integrating resources from clubs and universities (e.g., the “High School & University Partnership” model) to build a seamless physical activity network (32, 33).

### 4.3 Family engagement: reshaping parental cognition and health literacy

According to the evaluation indicators, family support has a weight of 0.2442, which is close to the weight of school support. This indicates that family support plays an important auxiliary role in the social support system for adolescent physical health promotion, mainly influencing adolescents through family sports atmosphere, family physical activity participation, and family sports-related consumption. Empirical results show that the family support dimension achieved a score of 75.40, lying at the threshold between “fair” and “good” overall. This indicates that family support for adolescent physical health promotion in China remains incomplete, and a stable and efficient support system has not yet been established. Although the overall situation has improved, cognitive biases and conceptual limitations remain core barriers constraining the effectiveness of family support. Within the family support dimension of the evaluation indicator system, family sports atmosphere has the highest indicator weight at 0.5278, which further confirms the importance of family sports beliefs. Survey data reveal that 70% of students are prohibited from participating in physical exercise after school, and 74% of parents never engage in sports with their children. Most parents regard physical activity only as a means to enhance physical fitness, ignoring its vital educational value in promoting cognitive development, rule awareness, and social competence (34). Such one-sided value perception directly leads to insufficient family support and a lack of companionship for adolescents’ sports participation. Existing studies confirm that parental emphasis on physical activity is significantly positively correlated with children’s physical activity levels (35). Parental attitudes and beliefs play a pivotal role in shaping adolescents’ health-related physical behaviors. Parental companionship and participation can significantly enhance children’s enjoyment and intrinsic motivation for exercise, strengthen parent–child communication and emotional bonding (36), and boost their self-identity and sense of belonging, thereby stimulating sustained enthusiasm for physical activity (37). Thus, transforming parental perceptions, strengthening family companionship, and building a supportive family sports environment represent critical pathways to improving the physical health of adolescents.

Implications for optimization:

(1) *Cognitive transformation*: Policies and media campaigns must target parental health literacy, emphasizing that physical activity promotes, rather than hinders, academic and cognitive development (38).
(2) *Role modeling*: Parents should act as active role models. Interventions like “Family Sports Day” can institutionalize family physical activity (39).
(3) *Holistic support*: Parents need to provide not only financial support (equipment, fees) but also emotional support (encouragement, companionship), which determines the sustainability of adolescents’ intrinsic motivation (40).

### 4.4 Community extension: revitalizing the grassroots support system

According to the evaluation indicators, community support has a weight of 0.0909, which is significantly lower than the other three support subjects. This reflects that the supporting role of communities in adolescent physical health promotion has not been effectively realized at the present stage. Community support mainly expands the venues for adolescent physical health promotion through various sports activities and sports organizations, and plays an extended role in promoting the physical health of adolescents. Empirical results show that community support is the weakest among all dimensions, with a score of only 60.35. This result highlights obvious structural gaps and insufficient functional supply at the grassroots governance level, which has become a critical weak link restricting the healthy development of adolescent physical health promotion. Existing theoretical and empirical evidence consistently shows that, as the main venue for adolescents’ out-of-school physical activity, the integrity of the community support system directly affects participation sustainability and health benefits. Anderson (41) pointed out that well-established community sports organizations can significantly improve adolescents’ physical activity participation, provide them with richer activity opportunities and social support, and thereby promote adolescent physical health development. Kurc and Leatherdale (42) found that regions with a higher proportion of community sports funding investment tended to have better adolescent physical activity participation and physical health status. In addition, Ettekal et al. (43) emphasized that scientific community sports guidance plays a key role in promoting adolescent physical health, and scientific training methods can significantly improve the athletic performance of adolescent athletes. Different from Western countries where communities serve as the core carrier of residents’ physical activity, communities in China have long undertaken heavy administrative tasks such as Party building, environmental governance, and public security management, resulting in a serious shortage of resources and energy available for public sports services. Coupled with the lack of specialized sports management institutions, insufficient funding investment, and a shortage of professional personnel, these combined problems have led to weak community sports support capacity, unbalanced supply structure, and low service quality. It is difficult to meet the growing needs of adolescents for physical health and physical activity, which also serves as a typical manifestation of unbalanced public service supply in the process of grassroots governance modernization.

Implications for optimization:

(1) *Resource integration*: Communities must leverage external resources by connecting with street offices and district sports bureaus to secure funding and personnel (44).
(2) *Facility accessibility*: The policy of opening public and school sports venues to the community must be strictly enforced to alleviate the venue shortage (45).
(3) *Service provision*: Communities should collaborate with NGOs and sports associations to organize accessible events (e.g., parent–child games) and utilize weekends and holidays to provide “doorstep” sports services (46, 47).

### 4.5 Research limitations and prospects

This study has certain limitations in indicator construction, data collection and expert selection, which can be further optimized in the future. First, this study employed the Delphi method to develop the indicator system, and adopted self-reported questionnaire assessments conducted by stakeholders including government officials, school administrators, community directors and parents. The high overlap between evaluators and respondents tends to cause self-report bias and social desirability effect, which may undermine construct validity. Future studies may introduce third-party independent evaluation to separate evaluation subjects from objects, thereby improving data objectivity. Second, the expert panel for indicator construction was composed exclusively of domestic university scholars, without the participation of international experts, policy-makers, adolescents and parents, leading to insufficient diversity and representativeness of expert opinions. A transdisciplinary, cross-sectoral and cross-cultural expert team should be established in the future to strengthen the comprehensiveness and applicability of the indicator system. Third, the indicator system in this study is relatively extensive, covering 4 primary indicators, 19 secondary indicators and 54 tertiary indicators, and the redundancy and multicollinearity of indicators were not tested. Subsequent studies can use factor analysis, correlation analysis and other methods to streamline and optimize indicators, so as to improve the discriminant validity and application efficiency of the evaluation system.

In conclusion, this study provides a preliminary exploration for the construction of relevant evaluation systems. Further improvements addressing the above deficiencies will significantly enhance the rigor and scientificity of the research, and provide a more reliable basis for practical application.

## Data Availability

The original contributions presented in the study are included in the article/supplementary material, further inquiries can be directed to the corresponding author.
